# Is heterologous prime-boost COVID-19 vaccination a concern or an opportunity for Ethiopia?

**DOI:** 10.3389/fpubh.2022.1046546

**Published:** 2023-01-26

**Authors:** Tesfaye Gelanew, Liya Wassie, Andargachew Mulu, Liya Wondwossen, Markos Abebe, Adane Mihret, Alemseged Abdissa

**Affiliations:** ^1^Armauer Hansen Research Institute, Addis Ababa, Ethiopia; ^2^Federal Ministry of Health, Addis Ababa, Ethiopia

**Keywords:** COVID-19, dose, Ethiopia, heterologous, homologous, vaccine, intramuscular, intradermal

## Introduction

Mass vaccination has become a pressing need to attenuate the ongoing coronavirus disease 2019 (COVID-19) pandemic. Despite considerable efforts, only one-third of the population in Ethiopia has been fully vaccinated so far (1), in stark contrast to the proportion of fully vaccinated people in developed countries. According to the Ethiopian Ministry of Health (MoH), 52.5 million COVID-19 vaccine doses have been administered in the country since the 30th of August 2022. With the increasing concern over waning vaccine-induced immunity and the continuous emergence of new variants of concern, including the Omicron variants (2, 3), countries with limited resources, such as Ethiopia, need to consider alternative strategies for the timely COVID-19 vaccination.

**Figure 1:**
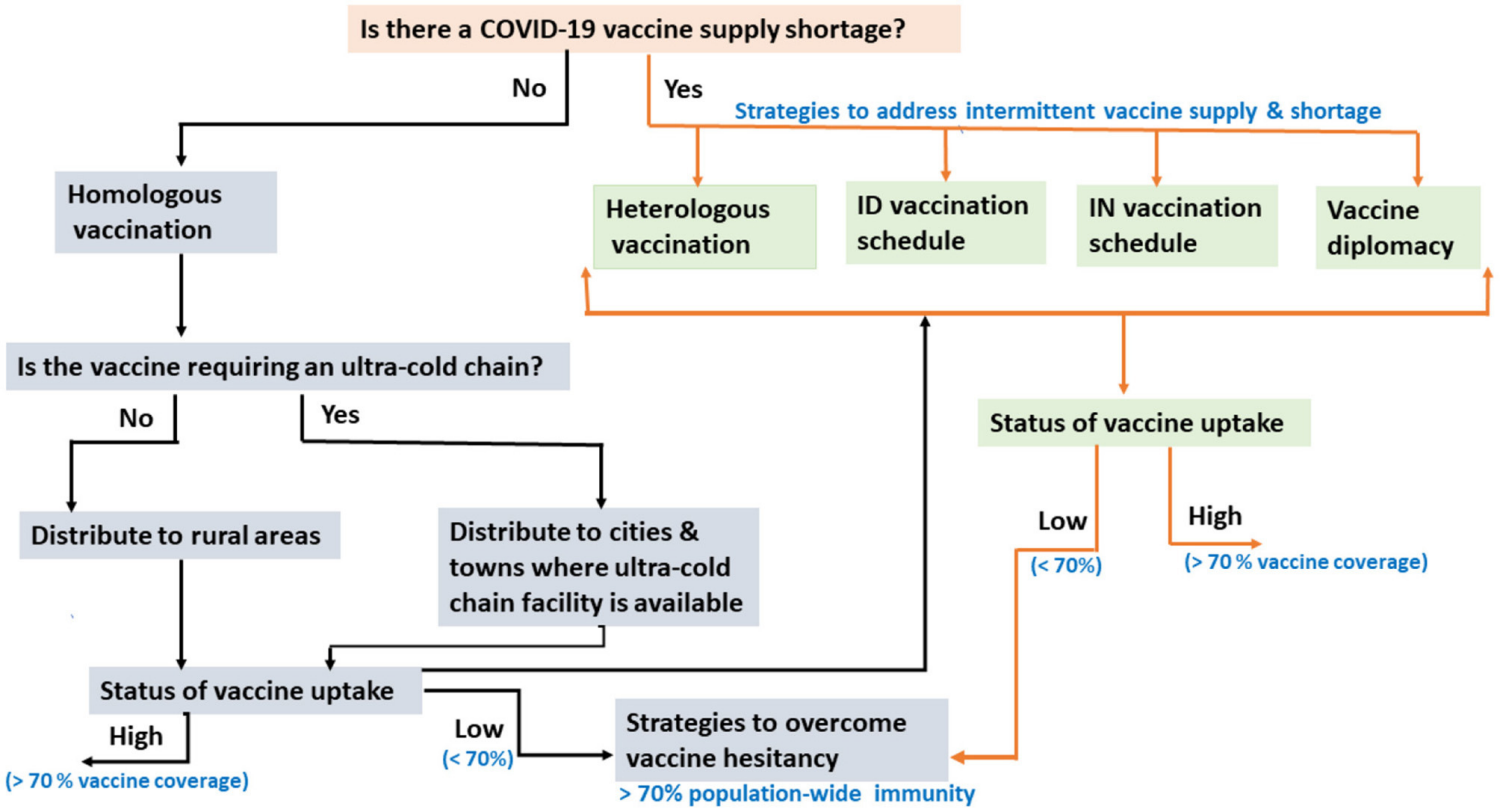
Flow chart showing the challenges of COVID-19 vaccination rollout in Ethiopia, with possible mitigation strategies. ID, Intradermal; IN, Intranasal.

Though Ethiopia is the second-most populous country in Africa, it has no local vaccine research and production capacity and remains highly dependent on imports, primarily through the support of the Global Alliance for Vaccines and Immunizations (GAVI). The COVID-19 vaccine rollout in Ethiopia has been constrained by two major factors: the unpredictable and intermittent COVID-19 vaccine supply and increased hesitancy to take the vaccine even when available due to the low-risk perception of COVID-19 (4). This can potentially be mitigated by switching to intradermal or intranasal vaccination, as these use a much smaller volume/fraction of the routine vaccine dose (5). Alternatively, heterologous vaccination regimes, with one vaccine type as a first dose and another as a second dose, could also be considered (5, 6). Besides the vaccine supply shortage and suboptimal vaccine uptake (7, 8), vaccine thermostability impacted the COVID-19 vaccine rollout in Ethiopia. In this opinion paper, we discuss COVID-19 vaccine rollout challenges in Ethiopia with possible mitigation strategies (Figure 1), with particular emphasis on a heterologous prime-boost vaccination approach as a means to curb the observed intermittent vaccine supply shortage in the country.

## Heterologous prime-boost vaccination schedule as a means to spare vaccine doses

Global initiatives such as the COVID-19 vaccines global access (COVAX), along with other key delivery partners such as UNICEF, have shown impressive progress in ensuring equitable access to COVID-19 vaccines. However, the solidarity initiatives encountered a challenge and thus were not optimal, mostly due to vaccine nationalization (9), increasing demand in developed countries, and the need for several subsequent booster doses for fully vaccinated individuals following the emergence of the Omicron variant (10). As a result, Ethiopia was forced to receive different COVID-19 vaccines from multiple suppliers that use different manufacturing platforms and doses. Some of the first COVID-19 vaccines received included the CoronaVac (Sinopharm, 13.7 million doses) from the Chinese government, the ChAdOx1 (Oxford–AstraZeneca, >6.99 million doses), the Ad26.COV2.S (Janssen, >34.5 million doses), BBIBP-CorV (~4 million doses) and the BNT162b2 (BioNTech-Pfizer, ~6.5 million doses) through the COVAX initiative, while the Ad26.COV2.S (>7.5 million doses) was provided through the African Union's African Vaccine Acquisition Trust (AVAT) initiative (Table 1).

**Table 1:** The sources, types and number of doses of COVID-19 vaccines received in Ethiopia as of 15/11/2022.

**No**	**Vaccine type**	**Source**			**Grand total**
		**Bilateral from The Republic of China (doses** ^*^ **)**	**COVAX**^a^ (doses^*^)	**AVAT**^b^ (doses^*^)	
1	ChAdOx1	_	6,979,440	_	6,979,440
2	Ad26.COV2.S	_	34,533,350	7,516,800	42,050,150
3	BBIP-CorV	13,700,000	3,890,400	_	17,590,400
4	BNT162b2	_	6,465,420	_	6,465,420
	Grand total	13,700,000	51,868,610	7,516,800	73,085,410
	By proportion	18.7%	71.0%	10.3%	100.0%

* Numbers given in relation to vaccine dose;

a COVAX, COVID-19 vaccines global access;

b AVAT, African Union's African Vaccine Acquisition Trust.

According to Ethiopia's national COVID-19 vaccination program (11), the recommended primary series two-dose regimens in 2021 were intramuscular (IM) administration of homologous BBIBP-CorV-BBIBP-CorV, homologous ChAdOx1-ChAdOx1, and homologous BNT162b2-BNT162b2, whereas Ad26.COV2.S vaccine was accepted as a single dose with an IM route of administration (12). The majority of frontline healthcare workers and elderly people received their first dose of ChAdOx1. However, the emergence of the Delta variant aggravated the global shortage of the ChAdOx1 vaccine (10). In addition, the production of ChAdOx1 was on hold in several countries due to concern over rare side effects, such as thrombosis, particularly in women (13). This resulted in an inadequate stock of the ChAdOx1 vaccine to administer the second booster dose to all who were vaccinated with the initial dose of the same vaccine and to boost all those individuals who had been primed with the ChAdOx1 vaccine. This subsequently forced the MoH to implement a heterologous prime-boost vaccination strategy. To mention a few, those healthcare workers who received ChAdOx1 as a first dose were later vaccinated with the Ad26.COV2.S vaccine as a second dose. Moreover, a third booster dosage would probably have required yet another type of COVID-19 vaccination for the elderly and healthcare professionals who had previously received two doses of ChAdOx1. This prompted us to question whether a heterologous prime-booster vaccination strategy for COVID-19 could become a concern or an opportunity in controlling the pandemic, particularly in the Ethiopian context.

The first-generation COVID-19 vaccines have been shown to be less effective against newly emerging or future re-emerging coronaviruses (2, 3). Therefore, it has become imperative to develop broad-spectrum anti-SARS-CoV-2 vaccines and therapeutics. In response to this demand, several second-generation pan-coronavirus vaccines have been developed, some in the preclinical stage and others in the clinical stage (14). Until pan-coronavirus vaccines become commercially available, heterologous prime-boost vaccination regimens with the best “mix and match” of currently available COVID-19 vaccines (Table 2), which could potentially result in the induction of breadth protective immunity against different variants of concerns, could be deployed as a valid alternative vaccination strategy (15, 16).

**Table 2:** Immunogenicity and reactogenicity of heterologous mix-and-match COVID-19 vaccination regimens in comparison to homologous vaccination for selected licensed COVID-19 vaccines.

**Prime vaccine brand**	**Boost vaccine brand**	**Target population**	**Immunogenicity**	**Reactogenicity**	**Reference**
ChAdOx1	BNT162b2	Adult healthcare workers	Superior immunogenicity	No difference in reactogenicity	(15)
Ad26.COV2.S	BNT162b2 or mRNA-1273	Healthy adults	No inferior immunogenicity	No safety concerns were identified	(16)
CoronaVac	ChAdOx1	Healthy adults older than 18 years	Superior immunogenicity	Not available	(17)
ChAdOx1	CoronaVac	Healthy adults older than 18 years	Lower immunogenicity compared to homologous ChAdOx1/ ChAdOx1 vaccinations	Not available	(17)
BNT162b2	ChAdOx1	Healthy adults aged 50 years and older	Higher immunogenicity compared with ChAdOx1/ ChAdOx1	Increased systemic reactogenicity in heterologous schedule	(18)
ChAdOx1	BNT162b2	Healthy adults aged 50 years and older	Higher immunogenicity compared with ChAdOx1/ ChAdOx1	No increased reactogenicity in the heterologous schedule	(18)
ChAdOx1	BNT162b2	Healthy healthcare workers	Higher immunogenicity	Tolerated reactogenicity	(19)
ChAdOx1	mRNA-1273	Healthy healthcare workers	Robust and strong immunogenicity	Relative higher reactogenicity, but well tolerated	(19)
Ad26.COV2.S	BNT162b2	Healthcare workers	Higher immunogenicity	Well-tolerated reactogenicity, which resolved in 48 h	(20)
Ad26.COV2.S	mRNA-1273	Healthcare workers	Higher immunogenicity	Well-tolerated reactogenicity, which resolved in 48 h	(20)

Such heterologous vaccination also seems to be applicable and productive compared to homologous vaccination in a pragmatic approach to COVID-19 vaccination (5), and other diseases such as HIV (6) and Ebola (21). On the contrary, short-term reactogenicity was higher with heterologous regimes than with homologous regimes (22). Results from studies in developed countries indicate the safety (tolerability) and effectiveness of heterologous prime-boost vaccination (13). Table 2 summarizes the immunogenicity and reactogenicity of heterologous prime-boost vaccination schedules with the selected licensed COVID-19 vaccines.

Although the highest antibody response is induced by mRNA vaccines as a heterologous second dose (22), the combination of heterologous prime-boost schedules with inactivated vaccines (like CoronaVac) and adenovirus-based vaccines (such as Ad26.COV2.S and ChAdOx1) is recommended as a feasible vaccine distribution in developing countries like Ethiopia (23) due to the extremely low or ultra-cold supply chain requirement (which is highly prone to product storage error) and cost of mRNA vaccines (24). Nevertheless, given the limited evidence from low-income countries, Ethiopia needs to assess the safety of different combinations of heterologous prime-boost vaccines to determine which mixing provides long-term protection for its population. When supply-chain distributions are limited, local data on immunogenicity and reactogenicity would give health policymakers more confidence to deploy a heterologous vaccination strategy in the future.

Administrating a third dose (booster, either homologous or heterologous) of vaccination has been shown to produce an immune response against different variants of concern, including the Delta and Omicron (25). Following the fourth COVID-19 wave in Ethiopia, presumedly related to the Omicron wave, the Ethiopian vaccination program has considered third booster dose vaccinations for the elderly, frontline healthcare workers, individuals with comorbid health conditions, and other high-risk populations. Given that 65% of the Ethiopian population is still not fully vaccinated, the mass administration of a third (booster) vaccine dose in Ethiopia is debatable (26) and we recommend against administering a third dose to population groups that are less exposed and at lower risk. If Ethiopia is forced to consider mass administration of a third dose in the future, third dose administration to individuals who have already been naturally infected and received two doses should be carefully considered only after (i) administering two doses to the remaining infection naive and unvaccinated people, (ii) administering the second dose to people primed with Ad26.COV2 and (iii) administering third-dose to high-risk populations and to people who received two doses of inactivated vaccines (e.g., CoronaVac) given inactivated vaccines have been found to be less immunogenic (22).

Overall, a heterologous vaccination schedules seem more practical in settings such as Ethiopia, where intermittent vaccine supply is prevalent and sustainable vaccination programs must be maintained. In addition, heterologous vaccination schedules can also reduce vaccine hesitancy (Figure 1) by providing alternative booster vaccination options (a different vaccine from the primed vaccine) to those individuals who experienced adverse events (AEs) after their prime (first dose) vaccination (16, 17).

## Fractional intradermal vaccine administration as a means to spare vaccine doses

Currently, all the licensed COVID-19 vaccines are administered by IM injection (12). IM tissue is known to bear transient antigen-presenting dendritic cells (APCs) (12). By contrast, the skin (dermis), which is targeted by intradermal (ID) delivery, contains a higher density of APCs such as dermal dendritic cells (DDCs) than muscle (27). In addition, the dermal lymphatic system is organized into several plexus systems, which aid in the efficient transport of vaccine antigens and APCs to the draining regional lymph nodes where further activation of B- and T-lymphocytes takes place (28). Studies have shown that ID delivery of a reduced or fractional vaccine dose (1/5th, 1/6th, or 1/10th of the standard dose of ChAdOx1, BNT162b2, and mRNA-1273) vaccines could elicit similar immunogenicity and reactogenicity to IM inoculation (28–30). Although these promising studies require validation in controlled randomized clinical trial studies in developing countries such as Ethiopia, ID immunization has great potential to be deployed as a vaccine dose-sparing strategy in the future. When there is a vaccine shortage, Ethiopia should also consider a fractional dosing scheme to save doses and achieve herd immunity quickly (Figure 1). As of 30 December 2022, 52.5 million doses of the COVID-19 vaccine have been administered in Ethiopia. If these doses had been administered with a one-fifth fractionation, the entire eligible population of the country could have already been fully vaccinated. In addition, ID administration may increase vaccine uptake among those who are hesitant about receiving a shot due to safety concerns about standard IM injection (30). One of the challenges regarding the large-scale implementation of ID administration is its technical difficulty (5). However, Ethiopia already has an adequate number of trained healthcare workers due to decades of routine administration of Bacillus Calmette–Guérin (BCG) and rabies vaccines, and they could successfully implement mass ID inoculation of COVID-19 vaccines.

## Intranasal vaccines as a strategy to spare vaccine doses

Protecting individuals from mucosal pathogens, including SARS-CoV-2, through vaccination may require the induction of both mucosal and systemic immune responses. However, existing IM vaccinations are meant to induce a systemic immune response without generating mucosal protection against viral replication and nasal shedding in the upper respiratory tract, leading to an asymptomatic or mildly symptomatic infection that can still transmit the virus (31). By contrast, intranasally (IN) administered COVID-19 vaccines have been shown in preclinical studies to induce mucosal protection and systemic immune responses (5, 32), some of which have entered different stages of clinical trials (31). If those IN vaccines are proven to be effective, they would be attractive alternatives to block the transmission of SARS-CoV-2. The fact that IN vaccines do not require needle injections makes them one of the most cost-effective vaccination strategies to enhance global vaccine coverage, particularly in resource-constrained countries (Figure 1). IN vaccines could also be given as a booster to those individuals who have already received their first dose through IM delivery.

## Vaccine diplomacy as a means to mitigate the vaccine supply shortage

Over 36 low-income countries have received COVID-19 vaccines from the Indian government through global vaccine diplomacy (33). However, Ethiopia has not yet exercised vaccine diplomacy as a valid alternative strategy to address its vaccine supply shortage. Thus, in addition to the existing vaccine supply platforms such as COVAX, Ethiopia should make use of vaccine diplomacy to meet its vaccine needs for fighting present and future pandemics (Figure 1).

## Considerations for using thermostable vaccines

Thermostable COVID-19 vaccines would be valuable to expedite vaccine rollout and thereby achieve the desirable population-wide immunity in low- and middle-income countries like Ethiopia (24). Despite efforts to develop thermostable COVID-19 vaccines, including DNA, inactivated, or protein subunit platforms, no approved thermostable vaccine that can be stored for prolonged periods at room temperature (20°C) has been developed until now (34). COVID-19 vaccine storage requirements currently range from ultra-cold temperatures (<-70°C) to refrigerator temperatures (2 to 8°C) (35). When different vaccines are available, their thermostability, alongside their efficacy and safety, should also be considered prior to their import and distribution across countries located in temperate regions. Unlike mRNA-based vaccines, which require stringent ultra-cold storage facilities, adenovirus-vectored vaccines (e.g., Ad26.COV2.S) are more compatible with the existing vaccine-cold chain system in Ethiopia and seem to be more attractive for achieving a population-wide immunity in rural and remote areas of the country (Figure 1).

## Local vaccine production

Technology transfer and waiver of vaccine technology intellectual property (IP) could help Ethiopia and other developing countries invest and develop their own manufacturing capabilities and capacities, which could help not only the ongoing COVID-19 pandemic but also future ones (36). At the moment, there is no existing local vaccine manufacturing facility in Ethiopia. As a result, waiving vaccine technology (IP) has little or no effect on meeting the current vaccine demand. However, there are ongoing discussions to produce vaccines for cholera, rabies, typhoid, yellow fever, meningitis A, and tuberculosis locally (37), and IP waivers could also aid in the realization of these ongoing plans.

## COVID-19 vaccine hesitancy and mitigation strategies

According to WHO, vaccine hesitancy has been one of the global threats to vaccine-preventable diseases (38), including COVID-19. Several studies in Ethiopia reported varying levels (ranging from 14.1 to 68.7%) of reluctance for COVID-19 vaccine uptake among healthcare workers (7, 39) and in rural and urban communities with a wide range of economic and social status (39–42). Some of the major factors reported for vaccine uptake hesitancy among healthcare workers included inadequate evidence and concerns over vaccines' safety, efficacy, and quality; a prior history of SARS-CoV2 infection; and the duration of vaccine effectiveness (7, 38, 41). Similarly, other factors such as gender (being female), younger age, the primary source of information (particularly social media), and safety, tolerability, and quality concerns over the available vaccines were reported among other communities, contributing to the acceptability of the uptake of the vaccine (39–42).

In addition to addressing the vaccine supply shortage and intermittent supply, identifying and understanding factors associated with vaccine hesitancy while also designing and implementing effective mitigation are essential to accelerate the uptake of COVID-19 vaccination and thereby achieve the WHO-recommended minimum vaccination coverage of at least 70% to achieve herd immunity in Ethiopia (Figure 1). It is therefore imperative that the MoH works with regional health authorities, the Ethiopian Food and Drug Authority (EFDA), and all other stakeholders to seriously address vaccine hesitancy and develop cost-effective national mitigation strategies using different platforms (41, 43). The mitigation strategies should include educational campaigns using multimedia (government media and social media), social mobilization and communication campaigns such as house-to-house youth campaigns, and institutionally-based (hospitals, schools, and universities) campaigns to increase individuals' level of compliance and confidence regarding the COVID-19 vaccine and thereby their decision to be vaccinated once vaccine becomes available (43). It is also important to note that vaccine hesitancy mitigation campaigns should be aligned with subsequent mass-vaccination campaigns. Without such collaborative efforts and innovative implementation strategies, population-wide immunity through mass vaccination campaigns may not be achieved, despite efforts to resolve vaccine supply shortages.

## Conclusion and future prospects

In conclusion, a heterologous prime-boost vaccination strategy should be considered a safe and reliable opportunity for African countries, including Ethiopia, to achieve the higher (>70%) national COVID-19 vaccination coverage, as recommended by WHO. At this time, we support the MoH initiative to administer a third vaccine dose to at-risk population groups. It is critical to generate local evidence on the immunogenicity and reactogenicity of various heterologous prime-boost combinations. Furthermore, technology transfer and investment in building its local human capacity and facility, in addition to collaboration and partnership efforts with international vaccine manufacturing platforms, are needed to meet the region's current and future epidemic and pandemic needs. While developing local vaccine manufacturing capacity, Ethiopia should consider vaccine diplomacy as a cost-effective supplement strategy for meeting its current COVID-19 vaccine demand.

## Author contributions

TG conceptualized the study, drafted and finalized the manuscript. TG and LWo contributed to national vaccination data retrieving. TG, LWa, AA, AMu, LWo, AMi, and MA contributed to reviewing. All authors approved the final version of the manuscript, contributed to preparing the manuscript, and their authorship meets the International Committee of Medical Journal Editors (ICMJE) criteria.
